# Typology of Public-Private Partnerships in Integrated Care: Evidence from a Municipality in Seoul, Korea

**DOI:** 10.5334/ijic.9045

**Published:** 2026-02-27

**Authors:** Il-Ho Kim, Cheong-Seok Kim, Bon Kim, Dong Hee Joo

**Affiliations:** 1 Seoul Public Health Research Institute, Seoul Metropolitan City Seoul Medical Centre, Korea; 2 Department of Sociology, Dongguk University-Seoul, Seoul, Korea; 3 Center for Collaborative Research on Population and Society, Dongguk University-Seoul, Seoul, Korea

**Keywords:** integrated care, public-private partnerships, Social Network Analysis, older adult care, disability care

## Abstract

**Objectives:**

Despite the significant advantages of public-private partnerships (PPPs), understanding of the organisational roles that shape partnership functioning within integrated care networks remains limited. This study investigated the structural characteristics of PPPs and identified organisational roles within an integrated care network in a Seoul district, Korea.Methods: Using the 2020 Integrated Care Network Survey, this study analyzed modularity, eigenvector centrality, and brokerage in 82 public and private organisations across five different types: district offices, public health centres, healthcare institutions, social welfare institutions, and community organisations.

**Results:**

Our results reveal that integrated care organisations are predominantly divided into two major networks: disability care and elderly care. The highest centrality in the elderly care network is occupied by the dementia relief centre and social welfare institutions, while the disability care network is dominated by public institutions, the Health and Welfare Cooperative, and a social service centre for the disabled. Regarding organisational roles, social welfare institutions function primarily as coordinators, representatives, and gatekeepers, whereas public institutions—including district offices, public health centres, and community service centres—serve as consultants and liaisons. Notably, public institutions assume more prominent roles and private organisations demonstrate less involvement in the disability care network compared to the elderly care network.

**Conclusion:**

These findings underscore the importance of defining clear organisational roles and interaction mechanisms between public and private sectors, offering valuable insights for policy design aimed at enhancing coordination, accountability, and sustainability within PPP-based integrated care systems.

## Introduction

Establishing public-private partnerships (PPPs) in integrated care has become a critical global policy initiative. Since the 1980s, PPP policies have gained prominence across social welfare states, driven partly by “the crisis of the welfare state” [1,2]. Government budget constraints, rising expenditures, and limited resources have rendered solely government-centred approaches insufficient for addressing public policy demands and delivering effective welfare services [3,4]. In response, many Western welfare countries have adopted PPP models within integrated care systems to provide comprehensive care meeting the diverse needs of older adults and individuals with disabilities [3,5,6]. The United States and England have implemented PPP models to develop infrastructure for planning, evaluating integrated care needs, and merging health and social services within joint networks [3,7]. Social democratic welfare states—including Canada, Denmark, Sweden, and Australia—have similarly adopted PPP approaches for healthcare and welfare service delivery [3,6]. South Korea has made substantial efforts to align with these policy objectives at both central and local government levels [8]. In 2019, the Ministry of Health and Welfare developed a foundational integrated care plan, while the Metropolitan Government has worked to establish a community-based integrated care system. This policy seeks to create an effective PPP framework through initiatives such as ‘Seoul Care’, a healthcare programme, and the ‘Dolbom SOS Centre’, providing one-stop customised care services. Through these efforts, the Seoul government aims to deliver integrated health and social welfare services to all citizens, particularly those facing urgent and unexpected care needs.

Despite these efforts, criticisms have emerged regarding the bifurcation between public resources and private organisations, as well as between public health centres and district offices. Domestic and international research indicates that establishing effective public-private collaboration networks has proven challenging [9,10,11,12,13]. Analysis of a pilot health and welfare office project revealed limited practitioner awareness of the need for public-private collaboration in these sectors [9]. Additionally, role distinctions between public and private workers were ambiguous, with insufficient coordination constraining project success. Other studies highlight segmentation in Korea’s health and welfare service delivery systems and inadequate government policies linking public and private organisations, limiting responsiveness to evolving elderly care needs [10]. While integrated community care attempts a comprehensive approach, health and welfare sector efforts often remain separate and lack effectiveness for meaningful integration [11,12]. Encouragingly, recent improvements in field practitioners’ attitudes and perceptions regarding health-welfare collaboration signal a potential shift toward more effective integration.

However, little is known about how organisational relationships are structured and roles distributed within collaborative networks for elderly and disability care. This study examines the structural and functional characteristics of integrated care networks involving public–private partnerships (PPPs), focusing on elderly and disability care services. Specifically, it aims to:

1. Identify whether elderly and disability care organisations form distinct subgroups within integrated care networks and determine which institutions serve as hubs in each domain.
2. Map key stakeholders within PPP networks and analyze their positions and interconnections.
3. Examine the roles of public and private organisations in integrated care partnerships, emphasizing the distribution of intermediary and coordinating functions.
4. Propose organisational roles and structural arrangements necessary to strengthen and sustain effective public–private collaboration in integrated care systems.

## Background

### The collaboration between public and private organisations

The evolution of social welfare systems fundamentally reflects the dynamic relationship between the private and public sectors. The PPP model has transitioned from government-centred, vertical governance toward a collaborative framework that emphasises cooperation and networking with diverse private stakeholders [14]. Historically, welfare development was predominantly state-driven, with private organisations largely following government directives. The literature demonstrates that PPPs offer substantial advantages by capitalizing on the complementary strengths and mitigating the limitations of both sectors [15]. The public sector faces inherent constraints—termed ‘government failure’—characterized by bureaucratic, uniform, and inefficient service delivery at scale [16]. Public organisations leverage their institutional capacity to provide resources through local care consultative bodies while fostering private sector engagement and promoting efficient resource utilization through coordinated partnerships. While the private sector contributes innovation and operational efficiency to service delivery, it encounters ‘market failure’ when market mechanisms govern public goods such as social welfare services [17]. For-profit organisations emphasise operational efficiency, cost-effectiveness, and scalability, whereas non-profit organisations prioritise social value, equity, and support for vulnerable populations [18,19]. The literature suggests these divergent motivations may either conflict—when efficiency-driven strategies undermine public welfare objectives—or complement one another, generating synergistic outcomes when operational efficiency aligns with social goals. The private sector’s flexibility, expertise, and managerial capacity at the frontline of welfare provision position it as an effective catalyst for welfare innovation. Particularly, private participation in public services introduces provider competition, enhancing service quality and promoting cost-effective delivery. Nevertheless, the commercialisation of care services can impede collaboration and coordination among local community groups through barriers in information sharing, beneficiary responsiveness, resource allocation, and joint planning.

Conversely, social economy organisations, including social enterprises and cooperatives, fulfill a vital intermediary function within PPPs [20,21]. These entities pursue dual social and economic objectives, bridging public institutions and private providers. They facilitate resource and information flows while fostering community engagement [20,21,22]. By strengthening coordination and governance within PPPs, social economy organisations reconcile divergent partner motivations and enhance the sustainable delivery of integrated care. Recognising and strategically positioning these organisations is therefore essential for achieving both efficiency and social equity within integrated care systems.

Gidron and colleagues (1992) classified four PPP models based on the burden of financial resources and the responsibility for service supply [21]. First, the government-dominant model, in which the government takes charge of both funding and delivery of care services. This model has long been recognised in the welfare states. Second, the third sector-dominant model, where non-profit or private organisations have significant discretion, either in managing programmes or through political processes during policy implementation or development (p.19). This model is known as the “market model”. Third, the dual model of state/non-profit relations, in which the government and non-profit sector share a responsibility for financing and providing services. Finally, a “collaborative-partnership model”, where the government is responsible for the financial burden, while the non-profit sector or private sector is in charge of providing services. With the introduction of the integrated care policy in 2018, the South Korean government adopted a collaborative model of supporting and managing private organisations through public finance, rather than directly providing care services [8]. This policy shift emphasises PPPs while reducing the government’s direct involvement in welfare service delivery. As local governments are front-line administrative agencies that provide comprehensive care services to residents, it is crucial to encourage non-profit and private organisations to participate and support cooperative initiatives in the integrated care network. Currently, public organisations maintain their functions as funders and planners in integrated care, while social welfare institutions and community organisations, also known as non-profit organisations, deliver care services as service providers [1,21].

Public-private partnerships (PPPs) are widely recognized in academic and business communities for their significance in delivering social welfare services. Without appropriate collaboration among PPP stakeholders in integrated care systems, administrative confusion and inefficiency can arise due to complicated procedures and potential service duplication between public and private entities [6,12,13]. Over recent decades, numerous studies have explored collaborative partnerships between public and private sectors across diverse countries [4,12,13]. Recent literature on integrated care networks has described PPP network structures, identified stakeholders, and measured effectiveness [12]. However, Korean studies reveal lacking collaboration between public and private organisations, and even among private organisations [23,24,25], attributed to performance-focused assessments, insufficient expertise, and inadequate institutional linkages. These findings indicate insufficient coordination between care organisations, resulting in service redundancy and gaps. While decades of research have examined various aspects of PPP models within welfare service networks, thorough examination of PPP frameworks, specific roles, and responsibilities remains limited. A pragmatic approach is needed to understand and refine the specific implementation status and role allocation required for effective PPPs.

Furthermore, the specific roles of public and private sectors vary across countries [13], depending on contextual problems and social environments. To achieve local community service integration, both sectors must strategically distribute roles according to each community’s specific context. Korean research indicates that public organisations predominantly assume coordinator roles within PPP models for welfare service networks, distinct from private sector functions [23,24]. However, another study suggests that public organisations also emphasize counseling and service provision similar to private organisations, creating difficulties in differentiating sectoral functions [25]. The absence of structured coordination systems for public-private agreements has resulted in formalized public management, raising concerns that conflicts may arise rather than collaboration. While debate intensifies over restructuring the public-focused service delivery system, the PPP approach for engaging private care organisations remains inadequately addressed. Against this background, brokerage concepts within SNA offer valuable frameworks for developing comprehensive and precise understanding of PPPs in integrated care contexts.

## Method

### Data source

Data for this study were derived from the 2020 Integrated Care Network Survey in Seoul, which is a locally representative cross-sectional survey. The survey period was conducted for approximately three months, from October 2020 to January 2021. This study investigated all the public-private organisations that participated in integrated care programmes in a community in Seoul, Korea. In early 2019, the research team established a Memorandum of Understanding (MOU) with the District A Office, stipulating that research findings would serve as foundational data for the district’s integrated care initiatives. The District A Office issued official cooperation letters to local community organisations to facilitate data collection and encourage survey participation. Information on integrated care organisations was obtained from the District A Office, which identified 82 public and private organisations across five categories: district offices (5), public health centers (4), healthcare institutions (17), social welfare institutions (37), and community organisations (19). The survey achieved a 100% response rate.

Collaborative relationships among community organisations were assessed by: 1) examining the existence of interaction, collaboration, and cooperative activities, and 2) quantifying collaboration strength using a 5-point Likert scale. To minimize COVID-19 influence, the survey employed self-administered questionnaires distributed via mail and supplemented with in-person visits, with official local government cooperation. Target interviewees included senior personnel engaged in care projects, as well as managers and middle managers familiar with organisational operations (IRB approval numbers: DUIRB-201912-05 and DUIRB-202103-03).

### Statistical analysis

**Figure 1:**
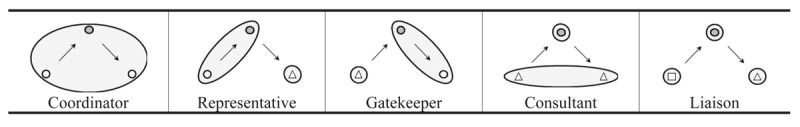
Five types of brokerage relationships. Note: Solid points represent actors, while the top point in each triad represents the broker. Circles indicate the boundaries of homogeneous groups (i.e., district offices, public health centres, healthcare institutions, social welfare institutions, and community organisations). Arrows indicate the direction of linkage according to the types of brokerage.

Social Network Analysis refers to the study of connection networks in which collaborative relationships between organisations are formed and spread [26]. It involves schematizing the network structure through a sociogram (Figure 1). It is a methodology that quantitatively analyzes the structure, interaction, and cohesion of a social network by modeling the relationship between individuals and groups as nodes and links. This analytical method can intuitively examine the collaborative patterns of public and private organisations participating in care projects for the elderly and the disabled in a community. SNA presents the form and pattern of collaboration between public and private care organisations in integrated care projects through quantified figures and relationship schematics. It can provide important policy implications for establishing an integrated care collaboration system. The purpose of this study was to investigate the structural characteristics and relationships within the network of 82 public and private care organisations in a community.

Here, we employed three methodological designs in the analysis of Social Network Analysis: 1) modularity, 2) eigenvector centrality, and 3) brokerage. First, we performed modularity analysis to assess the collaborative structure within the complex integrated care network. The quality of a community structure is evaluated by modularity, which is determined as the difference between the actual number of intra-community edges and the expected number of such edges. Modularity can decompose a complex network into several subnetworks, where nodes are densely connected. Secondly, we quantified the influence of organisations in the integrated care network by measuring eigenvector centrality. Finally, we analyzed brokerage patterns to identify the key actors and roles of public-private organisations in the integrated care network.

#### A. Network Modularity Indicators

Network modularity analysis is a quantitative technique used to detect communities clustered within an integrated care network. The ratio of the number of links within the community to the total connections is determined by assessing the probability that the number of links in the organisations is proportionate to their overall number of links. In other words, modular analysis can be used to quantify the extent to which each institution has more connections than expected by chance. Additionally, it can help classify these organisations into subgroups that share relatively close relationships within an integrated care network. Furthermore, organisations with collaborative partnerships are organised around a central institution that serves as a hub. Organisations with high connection strength tend to cluster and deploy together while rarely forming connections with organisations in other modules. In other words, a higher level of modularity suggests a more robust collaborative relationship among organisations within a group compared to between groups.

#### B. Eigenvector Centrality

Centrality measures play a crucial role in comprehending the integrated care network at the organisational level. In particular, eigenvector centrality is a specific indicator that measures the transitive influence of organisations in a network. The eigenvector score of an institution depends not only on its centrality score but also on the centrality of adjacent organisations. Connections from high-scoring organisations contribute more to an institution’s eigenvector centrality score than connections from low-scoring organisations. Eigenvector centrality refers to the ratio of importance that converges as the importance of each care institution is infinitely spread throughout the network. A high eigenvector score indicates that an institution is intricately linked with many significant organisations within a network.

#### C. Brokerage indicator

Brokerage analysis was conducted to identify intermediary roles of organisations within the integrated care network. A collaboration matrix was constructed from survey data to represent inter-organisational ties (1 = collaboration, 0 = none). Each organisation was classified into one of five sectors: district office, public health centre, social welfare institution, healthcare institution, and community organisation. Brokerage roles were calculated using the Gould and Fernandez (1989) procedure implemented in Netminer 4.0 [27]. Five brokerage types—Coordinator, Gatekeeper, Representative, Consultant, and Liaison—were identified based on sectoral membership of the broker and connected actors. The analysis examined the frequency and distribution of these roles to determine key boundary-spanning organisations facilitating intersectoral collaboration within the integrated care network [28]. This method provides insights into the structural position and functional influence of each organisation within the network [28]. More precisely, a broker is an intermediate organisation in a directed triad with directional connectivity that facilitates the development of PPPs and the delivery of integrated care services.

The first brokerage type is the coordinator, where all three care organisations form a directed triad chain within the same group. No partition separates these organisations, as the coordinator solely mediates information, resources, and service requests within a specified care group. The second type is the representative role, where an organisation receives information, service requests, resources, and project planning from organisations within its group and delivers them to organisations in different groups. Third, the gatekeeper maintains partnerships with key organisations strongly connected to both external and internal groups, serving as a mediator by transferring information, service requests, and resources from partnerships with other groups while facilitating external communication for their organisations. Fourth, the consultant serves as a mediator fostering collaborative relationships between two organisations within the same group, while remaining separate from these organisations and belonging to a distinct group. Finally, the liaison connects two distinct organisations while remaining impartial, belonging to an external group separate from both the initiator and receiver.

## Results

**Figure 2:**
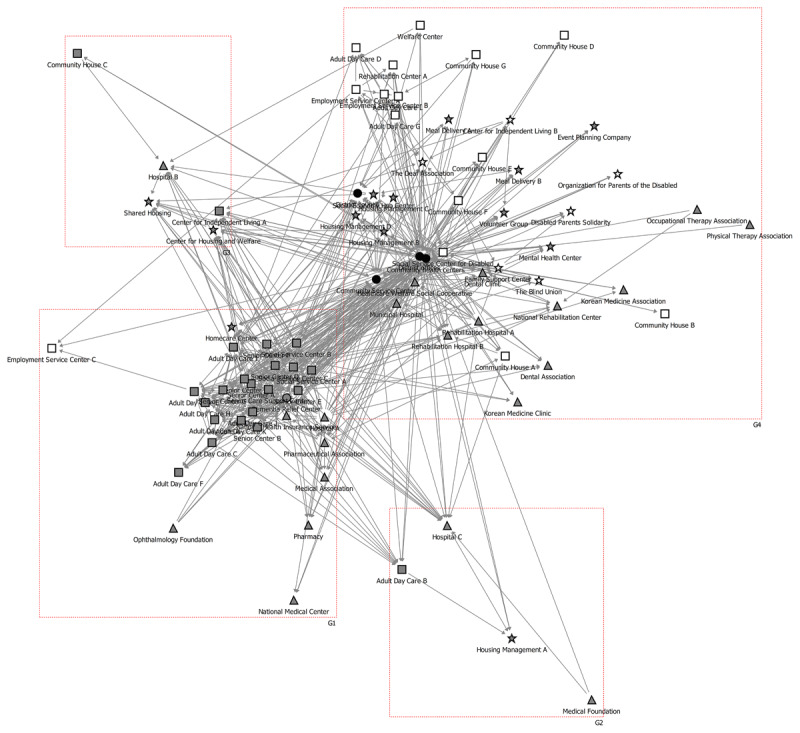
Modular structures of total community organisations of integrated care in a municipality in Seoul. Notes: 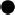 : District offices & Public health centres, 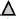 : Healthcare institutions, 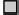 : Social welfare institutions, 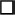 : Social welfare institutions for disabled, 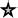 : Community organisations, 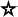 : Community organisations for disabled.

Figure 2 presents the collaborative network typologies derived from modularity analysis of all care-related organisations within the study area, revealing subgroup agglomeration structures based on collaboration intensity. Two primary collaborative networks emerged, centred on elderly and disability care organisations. Group G1 comprises 29 organisations (35.4%) focused on elderly care, including one dementia relief centre, twenty adult day care and social service centres, seven public and private healthcare institutions, and one community organisation.

Group G4 constitutes a disability care subgroup of 44 organisations, representing 52% of all organisations. These are evenly distributed across sectors: three district organisations (district office, community service centre, and district council), twelve healthcare institutions, twelve social welfare institutions, and sixteen community organisations.

Table 1 presents the five organisations with the highest eigenvector centrality across three network configurations: the overall collaborative network of 82 care-related organisations, the elderly care network, and the disability care network. In the overall collaborative network, the top five organisations are district offices, social service centre D, the dementia relief centre, community service centres, and public health centres. This indicates that, except for social service centre D, the four highest-ranking positions are occupied by public organisations, reflecting their prominent collaboration and interaction within the public-private partnership network.

**Table 1:** Five organisations with the highest Eigenvector Centrality.


	TOTAL		ELDERLY CARE NETWORK		DISABILITY CARE NETWORK	

1	District Offices	0.285	Dementia Relief Centre	0.291	District offices	0.405

2	Social Service Centre D	0.242	Social Service Centre D	0.284	Health & Welfare Cooperative	0.313

3	Dementia Relief Centre	0.226	Senior Centre A	0.284	Public Health Centres	0.293

4	Community Service Centre	0.220	Senior Centre C	0.260	Social Service Centre for Disabled	0.285

5	Public Health Centres	0.217	Senior Centre D	0.255	Community Service Centre	0.241


Within the elderly care network, the top five organisations ranked by eigenvector centrality are the dementia relief centre, social service centre D, and senior centres A, C, and D. In the disability care network, the highest-ranking organisations are district offices, health and welfare cooperatives, public health centres, social service centres for the disabled, and community service centres.

### Comparing the brokerage roles between networks for the elderly and the disabled

Brokerage analysis of the elderly and disability care collaborative networks yielded the following results.

### Coordinator

In the elderly care network, eleven social welfare agencies functioned as coordinators, including one social service centre, five senior centres, four adult day care centres, and one senior care support centre, each with coordinator scores of 30 or higher. These social welfare institutions facilitate coordination among organisations within the same sector through caregiving information exchange, beneficiary service requests, case management, resource sharing, and joint planning. Notably, neither healthcare institutions nor community organisations fulfilled coordinator roles (Table 2).

**Table 2:** Coordination scores for the networks for the elderly and disabled care.


	ELDERLY CARE NETWORK			DISABILITY CARE NETWORK		

1	Senior Centre C	D	80	Social Economy Hub Centre	E	11

2	Social Service Centre D	D	63	Adult Day Care L	D	9

3	Adult Day Care J	D	53	Adult Day Care G	D	8

4	Senior Centre A	D	52	Employment Service Centre B	D	2

5	Senior Centre D	D	40	Health & Welfare Cooperative	C	1

6	Senior Care Support Centre	D	40	Housing Management B	E	1

7	Senior Centre F	D	37	Community House F	D	1

8	Adult Day Care E	D	36	National Rehabilitation Centre	C	1

9	Senior Centre B	D	31	District Offices	A	0

10	Adult Day Care K	D	31	Public Health Centres	B	0

11	Adult Day Care A	D	30	Community Service Centre	A	0


**Figure 3:**
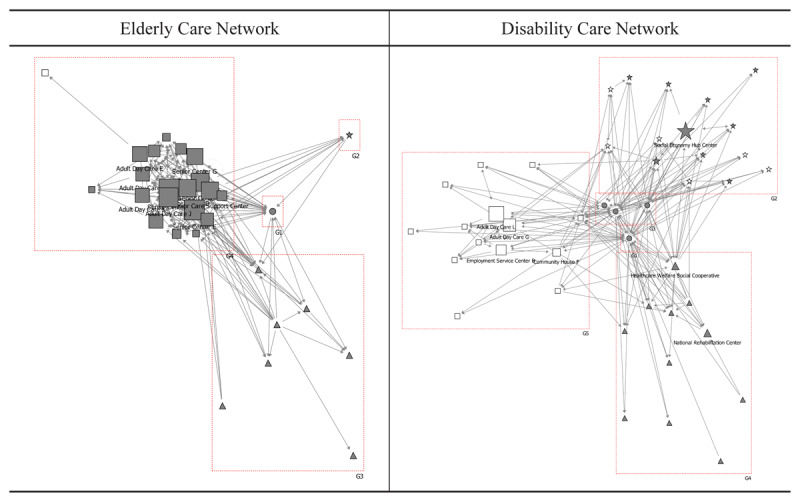
Coordination role in the collaborative networks for the elderly and disabled care. Notes: 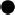 : District offices & Public health centres, 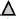 : Healthcare institutions, 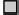 : Social welfare institutions, 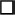 : Social welfare institutions for disabled, 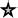 : Community organisations, 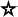 : Community organisations for disabled.

Conversely, within the disability care network, only a social economy hub centre and two adult day care centres served as coordinators, with scores ranging from 8 to 11, indicating limited coordinator engagement (Figure 3).

### Representative

**Figure 4:**
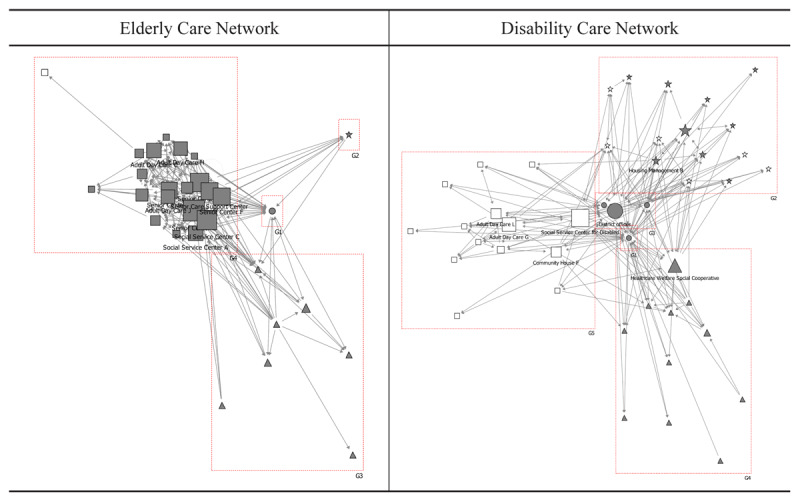
Representative roles in the collaborative networks for the elderly and disabled care. Notes: 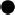 : District offices & Public health centres, 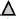 : Healthcare institutions, 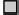 : Social welfare institutions, 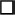 : Social welfare institutions for disabled, 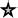 : Community organisations, 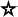 : Community organisations for disabled.

In the elderly care network, six social welfare institutions achieved representative intermediary scores of 10 or higher: two social service centres, three senior centres, and one senior care support centre. These institutions established working relationships as intermediaries between healthcare and social welfare institutions, either through public organisation facilitation or through direct mediation with healthcare institutions Figure 4.

In the disability care network, four organisations attained representative scores of 10 or higher: a social service centre for the disabled, district offices, two adult day care centres, and a health and welfare cooperative. These organisations, affiliated with various entities, established working relationships with district offices, mediated between organisations across different domains, and created direct connections spanning multiple fields. However, private organisations assumed representative roles less prominently in the disability care network compared to their counterparts in elderly care collaborations (Table 3).

**Table 3:** Representative scores in the collaborative networks for the elderly and disabled care.


	ELDERLY CARE NETWORK			DISABILITY CARE NETWORK		

1	Social Service Centre D	D	37	Social Service Centre for Disabled	D	39

2	Social Service Centre C	D	28	District Offices	A	21

3	Senior Centre A	D	15	Adult Day Care G	D	10

4	Senior Care Support Centre	D	12	Health & Welfare Cooperative	C	10

5	Senior Centre E	D	12	Social Economy Hub Centre	E	8

6	Senior Centre C	D	10	Adult Day Care L	D	5

7	Adult Day Care C	D	8	Community House F	D	5

8	Social Service Centre A	D	8	Housing Management B	E	3

9	Adult Day Care J	D	7	Employment Service Centre B	D	1

10	Adult Day Care H	D	7	National Rehabilitation Centre	C	1


### Gatekeeper

**Figure 5:**
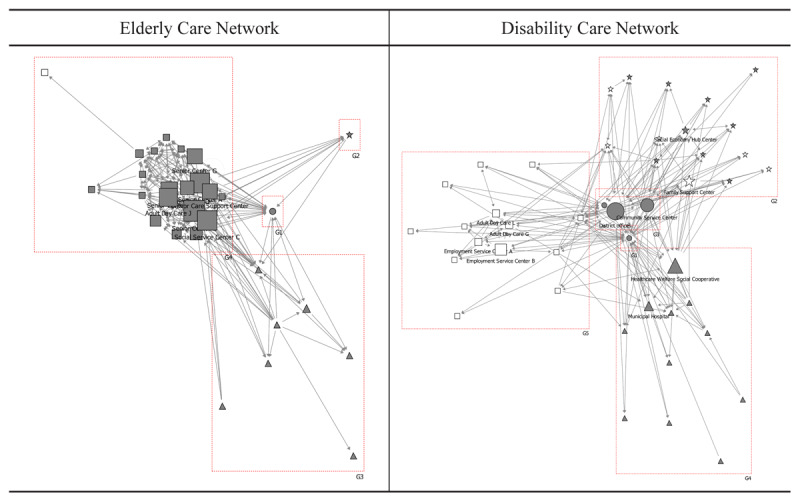
Gatekeeping roles in the collaborative networks for the elderly and disabled care. Notes: 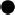 : District offices & Public health centres, 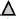 : Healthcare institutions, 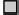 : Social welfare institutions, 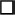 : Social welfare institutions for disabled, 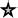 : Community organisations, 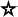 : Community organisations for disabled.

In the elderly care network, seven social welfare institutions achieved gatekeeping scores of 10 or higher: two social service centres, three senior centres, one adult day care centre, and one senior care support centre. These institutions functioned as gatekeeping intermediaries between private and public institutions, healthcare organisations, and community organisations, receiving care-related information from external entities and disseminating it within their sectors, thereby facilitating public-private communication Figure 5.

Within the disability care network, only two institutions—district offices and the health welfare cooperative—attained gatekeeping scores of 10 or higher. Although social welfare institutions assumed more prominent gatekeeping roles in the elderly care network compared to the disability care network, their overall involvement remained limited in both networks (Table 4).

**Table 4:** Gatekeeping scores in the collaborative networks for the elderly and disabled care.


	ELDERLY CARE NETWORK			DISABILITY CARE NETWORK		

1	Social Service Centre D	D	39	District Offices	A	22

2	Social Service Centre C	D	20	Health & Welfare Cooperative	C	16

3	Senior Centre A	D	20	Community Service Centre	A	7

4	Adult Day Care J	D	15	Employment Service Centre B	D	6

5	Senior Centre C	D	11	Family Support Centre	E	6

6	Senior Care Support Centre	D	10	Municipal Hospital	C	2

7	Senior Centre F	D	10	Adult Day Care G	D	1

8	Senior Centre D	D	8	Social Economy Hub Centre	E	1

9	Social Service Centre A	D	5	Adult Day Care L	D	1

10	Senior Centre B	D	4	Employment Service Centre A	D	1


### Liaison

**Figure 6:**
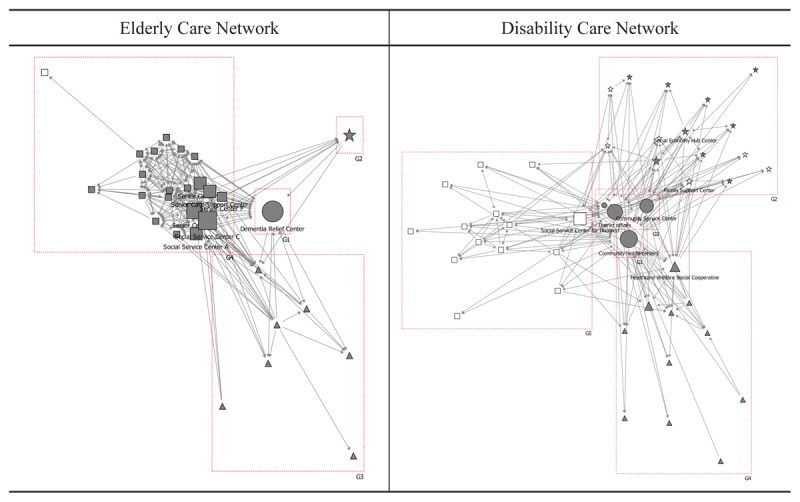
Liaison roles in the collaborative networks for the elderly and disabled care. Notes: 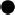 : District offices & Public health centres, 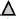 : Healthcare institutions, 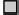 : Social welfare institutions, 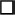 : Social welfare institutions for disabled, 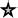 : Community organisations, 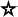 : Community organisations for disabled.

In the elderly care network, the dementia relief centre achieved a notably high liaison score of 88, playing a crucial role primarily connecting social welfare and healthcare institutions. However, its liaison function for social welfare centres remained limited. In the disability care network, public health centres, district offices, and community service centres attained the highest liaison scores at 415, 377, and 58 respectively, indicating that public institutions fulfilled more critical liaison roles in disability care compared to elderly care networks. Conversely, private organisations—specifically a social service centre for the disabled and the health and welfare cooperative—achieved higher liaison scores (39 and 26 respectively) than their elderly care counterparts (Table 5). These organisations functioned as intermediaries connecting social service centres for the disabled, healthcare institutions, and community organisations Figure 6.

**Table 5:** Liaison scores in the collaborative networks for the elderly and disabled care.


	ELDERLY CARE NETWORK			DISABILITY CARE NETWORK		

1	Dementia Relief Centre	B	88	Public Health Centres	B	415

2	Social Service Centre C	D	6	District Offices	A	377

3	Social Service Centre D	D	5	Community Service Centre	A	58

4	Senior Centre A	D	3	Social Service Centre for Disabled	D	39

5	Senior Care Support Centre	D	3	Health & Welfare Cooperative	C	26

	Social Service Centre A	D	3	Municipal Hospital	C	2

7	Homecare Centre	E	3	Housing Management B	E	2

8	Senior Centre E	D	1	Family Support Centre	E	1

9	Adult Day Care J	D	0	Social Economy Hub Centre	E	1

10	Senior Centre C	D	0	Employment Service Centre B	D	0


## Discussion

### Complexity of collaborative partnerships in integrated care

This study employed network analysis techniques—including modularity analysis, centrality analysis, and brokerage analysis—to examine interaction processes between public and private organisations within the integrated care landscape. Specifically, it investigated the complexity of collaborative public-private partnerships (PPPs) and organisational roles within integrated care networks. First, modularity mapping revealed two distinct collaborative networks: elderly care (n = 29) and disability care (n = 44). Second, eigenvector centrality analysis identified public institutions as occupying central roles within care networks, with certain private organisations ranking second. Third, brokerage analysis demonstrated distinct mediator roles between public and private organisations across both networks. Private organisations, including social service centres and community organisations, functioned primarily as coordinators, representatives, and gatekeepers, while public institutions exerted substantial influence as consultants and liaison intermediaries.

#### 1. Two different networks

Modularity analysis reveals that integrated care organisations are divided into separate elderly and disability networks, underscoring insufficient collaboration between these domains. In Korea, individuals with disabilities transition from the disability support system to the long-term elderly care insurance system at age 65. Ensuring smooth transitions requires disability and elderly care organisations to collaborate through information and resource exchange, service coordination, and joint planning. Previous research identifies that individuals aging with disabilities face heightened risk of dual jeopardy from both disability and age [29], encountering challenges in accessing appropriate healthcare and social services due to social exclusion and discrimination. However, the current integrated care network predominantly focuses on elderly populations, neglecting the multifaceted needs of people aging with disabilities and potentially disregarding their autonomy and unique requirements.

#### 2. Centrality comparison

The eigenvector centrality analysis reveals both commonalities and distinctions between the elderly care and disability care networks. A key commonality is the highest eigenvector centrality observed in public institutions, including dementia relief centres, district offices, public health centres, and community service centres. Wasserman and Faust (1999) define eigenvector centrality as quantifying the strength rather than the number of organisational connections within a network [30]. Our results indicate that public institutions occupy central positions in fostering PPPs across both care networks. Despite the recent transition toward pluralistic welfare service delivery that has diminished direct public sector roles and emphasized public-private collaboration [26], our findings demonstrate that public institutions remain the most active in cooperation and linkage activities. They exert the greatest network influence by connecting with high-ranking organisations, maintaining their significant position through involvement in legal framework development, policy planning and implementation, and PPP formation [31].

Notably, certain private organisations ranked second-highest in eigenvector centrality within both networks. This outcome reflects the network’s marketization objectives and the promotion of public-private collaboration [32,33]. Following Korea’s 1993 amendment to the Elderly Welfare Act permitting private sector participation [34], a division of responsibilities emerged: public organisations manage budgets and policy planning, while private organisations such as social welfare institutions develop and deliver government-approved care services. Social welfare institutions, registered as corporate legal entities, are particularly essential for service provision through government subsidies. Consistent with previous research, our findings suggest that the Health and Welfare Cooperative, a social economy organisation, functions as a core actor within the disability care network [12,31]. Meghan Cope (2001) argues that social economy organisations engage in political and social advocacy by establishing vital social connections and providing ground-level interpretation of welfare policies [35]. Scholars characterize these organisations as practical entities that enhance the publicity and accessibility of community welfare services while responding to social service marketisation [17,35,36]. These organisations appear to fulfill dual roles as implementers and advocates, positioning themselves at the nexus between public institutions and private organisations.

#### 3. Brokerage in collaborative networks for the elderly and disabled care

Brokerage analysis reveals that public and private organisations fulfill distinct mediator roles across elderly and disability care networks. Public institutions exert substantial influence as consultants and liaison intermediaries, while private organisations—including social service centres and social economy organisations—achieve the highest mediator scores as coordinators, representatives, and gatekeepers. Given the fundamental differences between public and private organisations, fostering cooperation requires adherence to principles of loyalty, mutual benefit, interdependence, and openness. This study confirms that public institutions serve as leading hubs for smooth cooperation and efficient decision-making by bridging and coordinating diverse organisations within care networks.

Schwartz (1977) emphasizes that liaison organisations are essential to network cohesion; their removal destroys network connectivity [37]. As representatives performing liaison roles, public institutions expend greater effort on administrative duties, public communications, conflict resolution, and coordination with private organisations. Conversely, Tushman and Katz (1980) indicate that private organisations effectively perform tasks beyond mere information or resource transmission, promoting exchanges between internal and external organisations through training, guidance, and socialization within their groups [38]. Our findings suggest that balanced public-private roles can simultaneously achieve accountability, fairness, equity, openness, and efficiency in care service provision.

This study demonstrates differential mediator role strength between disability and elderly care networks. Gatekeeper, representative, and liaison roles of public organisations are more prominent in disability care networks, reflecting Korea’s historical disability care development. First, disability care evolved primarily through national support for disability facilities, culminating in the 1961 Life Protection Law [39]. Private organisations only began establishing public-private consultation bodies for Community-Based Rehabilitation (CBR) in 2018. CBR programmes aim to integrate fragmented disability health, medical, and welfare services [40], yet legislation supporting community-based disability care was not introduced until December 2020, leaving insufficient time for collaborative network development. Second, factors hindering independent living vary by disability type, yet support for complex disabilities remains limited amid ineffective governmental coordination. Third, disability care predominantly comprises activity support services, initiated by the self-advocacy movement marking a paradigm shift from ‘rehabilitation’ to ‘independence’ [41]. However, local welfare networks connecting and enhancing social support for disabled individuals remain underdeveloped. While disability care services were integrated into local transfer projects, strict adherence to central government guidelines constrains PPP establishment flexibility.

A notable finding from this study highlights the diverse roles of social economy organisations and non-profit entities, including the Health & Welfare Cooperative, Social Economy Hub Centre, and welfare service institutes. Consistent with international research, these organisations emerge as primary stakeholders, collectively assuming responsibilities as coordinators, representatives, gatekeepers, and liaison intermediaries [12,20,21,33,35,42]. The Health & Welfare Cooperative exemplifies how social economy organisations operate under principles distinct from traditional businesses, pursuing dual non-profit and profit objectives while supporting community welfare facilities. Serving as a central hub in the disability care network, it facilitates coordination, resource flows, and community engagement. By occupying these pivotal positions, social economy organisations mediate between public and private actors, balance efficiency and equity objectives, and sustain integrated care delivery across elderly and disability care networks [20,35,42].

This study reveals that community organisations and healthcare institutions exhibit weak leadership roles within integrated care networks. These organisations demonstrate neither high eigenvector centrality nor significant intermediary functions. This marginal positioning may compromise network performance, particularly when these entities participate in case management and information sharing across varied organisational levels. Alter (2009) identified key motivations for establishing social welfare service networks: resource acquisition, organisational growth, enhanced competitiveness, and cost reduction [43]. In integrated care, collaborative pooling of diverse assets across organisations addresses the complex needs of care recipients. Individuals requiring care need coordinated assistance—including nursing, healthcare, housing, and daily living support—to remain within their communities. This necessitates efficient PPP network operations among diverse local organisations. Therefore, identifying and nurturing local community organisations and healthcare institutions, while encouraging their active network participation, is essential.

Based on these findings, several actionable recommendations are proposed for policymakers and practitioners to strengthen integrated care partnerships through effective public–private collaboration. First, establishing clear responsibilities and communication channels between public and private organisations is essential to enhance accountability and minimize service duplication. Second, social economy and non-profit organisations require institutional and financial support to reinforce their bridging and coordinating functions within care networks. Third, community and healthcare organisations need tailored capacity-building programmes, incentives, and appropriate intermediary role assignments to facilitate active engagement in integrated care partnerships. Fourth, decentralised and adaptive policy frameworks should enable municipalities to tailor PPP models to local contexts and resources. Finally, formal agreements or memoranda of understanding (MOUs) can clarify decision-making authority and accountability pathways across organisations. These findings underscore the importance of strengthening intermediary capacities and clearly defining organisational roles to enhance coordination, accountability, and sustainability in PPP-based integrated care systems.

This study provides empirical evidence on public-private roles in integrated care through a comprehensive regional survey in Korea, contributing to understanding mediator functions within such networks. However, transferring these findings to other countries requires careful consideration of local contexts. As PPPs have evolved from simple contractual mechanisms into policy- and institution-based collaborative frameworks, their applicability varies across nations depending on institutional arrangements, integrated care system structures, and the nature of public–private relationships. Further comparative research across multiple countries is needed to elucidate the dynamics of public–private collaboration in integrated care systems.

## Conclusion

Using social network analysis, this study addresses critical gaps in understanding integrated care network structures, mediator roles of public and private organisations, and associated challenges. First, modularity analysis reveals that integrated care organisations are separated into distinct elderly and disability networks, indicating insufficient cross-sector collaboration. Second, public institutions exhibit the highest eigenvector centrality, followed by social welfare institutions and social economy organisations. Third, mediator roles differ markedly between networks: public institutions primarily serve as consultants and liaisons, while private organisations—including social welfare institutions and social economy organisations—function as coordinators, representatives, and gatekeepers. Notably, public organisations play a more prominent role in disability care networks, whereas private sector involvement remains comparatively limited in this domain.

These findings underscore several imperatives. Private organisations require support and encouragement to assume mediating roles within disability care systems. Social economy organisations need assistance to foster independence and sustainable growth, promoting social values through community collaboration. Importantly, social economy organisations function not merely as service providers but as collaborative platforms bridging public and private sectors. Effective governance with clearly defined intermediary and facilitator roles is essential for promoting communication, coordination, and collaboration within PPPs.
